# NF-κB-Mediated Inflammation in the Pathogenesis of Intracranial Aneurysm and Subarachnoid Hemorrhage. Does Autophagy Play a Role?

**DOI:** 10.3390/ijms19041245

**Published:** 2018-04-19

**Authors:** Elzbieta Pawlowska, Joanna Szczepanska, Karol Wisniewski, Paulina Tokarz, Dariusz J. Jaskólski, Janusz Blasiak

**Affiliations:** 1 Department of Orthodontics, Medical University of Lodz, 92-216 Lodz, Poland; elzbieta.pawlowska@umed.lodz.pl; 2 Department of Pediatric Dentistry, Medical University of Lodz, 92-216 Lodz, Poland; joanna.szczepanska@umed.lodz.pl; 3 Department of Neurosurgery and Neurooncology, Medical University of Lodz, Barlicki University Hospital, Kopcinskiego 22, 90-153 Lodz, Poland; karol.lek@poczta.fm (K.W.); dariusz.jaskolski@umed.lodz.pl (D.J.J.); 4 Department of Molecular Genetics, Faculty of Biology and Environmental Protection, University of Lodz, 90-236 Lodz, Poland; paulina.tokarz@biol.uni.lodz.pl

**Keywords:** intracranial aneurysm, subarachnoid hemorrhage, inflammation, NF-κB, autophagy, early brain injury, delayed brain injury

## Abstract

The rupture of saccular intracranial aneurysms (IA) is the commonest cause of non-traumatic subarachnoid hemorrhage (SAH)—the most serious form of stroke with a high mortality rate. Aneurysm walls are usually characterized by an active inflammatory response, and NF-κB (nuclear factor kappa-light-chain-enhancer of activated B cells) has been identified as the main transcription factor regulating the induction of inflammation-related genes in IA lesions. This transcription factor has also been related to IA rupture and resulting SAH. We and others have shown that autophagy interacts with inflammation in many diseases, but there is no information of such interplay in IA. Moreover, NF-κB, which is a pivotal factor controlling inflammation, is regulated by autophagy-related proteins, and autophagy is regulated by NF-κB signaling. It was also shown that autophagy mediates the normal functioning of vessels, so its disturbance can be associated with vessel-related disorders. Early brain injury, delayed brain injury, and associated cerebral vasospasm are among the most serious consequences of IA rupture and are associated with impaired function of the autophagy–lysosomal system. Further studies on the role of the interplay between autophagy and NF-κB-mediated inflammation in IA can help to better understand IA pathogenesis and to identify IA patients with an increased SAH risk.

## 1. Introduction

On average, one in 25 persons can be at risk of intracranial aneurysm (IA), a cerebrovascular disorder which may lead to devastating subarachnoid hemorrhage (SAH) when ruptures occur [1]. In turn, half of SAH cases result in death in a few weeks after its occurrence, and less than half of the remaining patients is able to conduct a normal life. However, the pathogenesis of IA and the events preceding its rupture are poorly known. Therefore, efficient IA prevention and detection can decrease the number of life-threatening cases of IA-related SAH. Neurosurgical clipping and endovascular coiling treatment of IA to prevent its rupture are invasive procedures, which are associated with a significant complication rate. Moreover, IAs that have been coiled are at the risk of recanalization [2,3]. On the other hand, a “watch and see” strategy is often emotionally unacceptable to those patients with a constant fear of impending bleeding. Hence, studies on the molecular mechanism of IA formation, development, and rupture are justified. Inflammation is usually detected in IA walls and it can also contribute to the remodeling of the cerebral vessel wall associated with IA rupture. Many proteins control inflammation, including the transcription factor NF-κB (nuclear factor kappa-light-chain-enhancer of activated B cells), which is important not only for inflammatory processes, but also for several vital functions of the cell, including proliferation.

NF-κB interacts with numerous proteins, including those important for autophagy, a process of recycling of no longer needed or damaged cellular components. Autophagy is implicated in the pathogenesis of many serious diseases, including cerebral disorders such as cerebral ischemia and resulting brain injury [4], traumatic brain injury [5], intracerebral hemorrhage [6], and others. Some studies show that IA formation and its rupture can be related to the expression of certain autophagy proteins [7,8]. Therefore, it is justified to explore the link between IA/SAH, inflammation, and autophagy, as an element of IA/SAH pathogenesis and to assess its potential in the prevention, diagnosis, and therapy of IA/SAH.

## 2. Intracranial Aneurysm

Saccular IA (further called IA) is a stable, localized dilation (outpouching) of the arterial wall resulted from the weakening of its muscular layer [9,10]. Such IAs are fairly common and occur in a considerable proportion (up to 10%) in some populations [11]. Intact (unruptured) IA is mostly asymptomatic and often discovered by chance. However, it carries a potential risk of rupture and fatal SAH. Although the severity of SAH varies, its overall mortality rate exceeds 50% [11].

**Figure 1:**
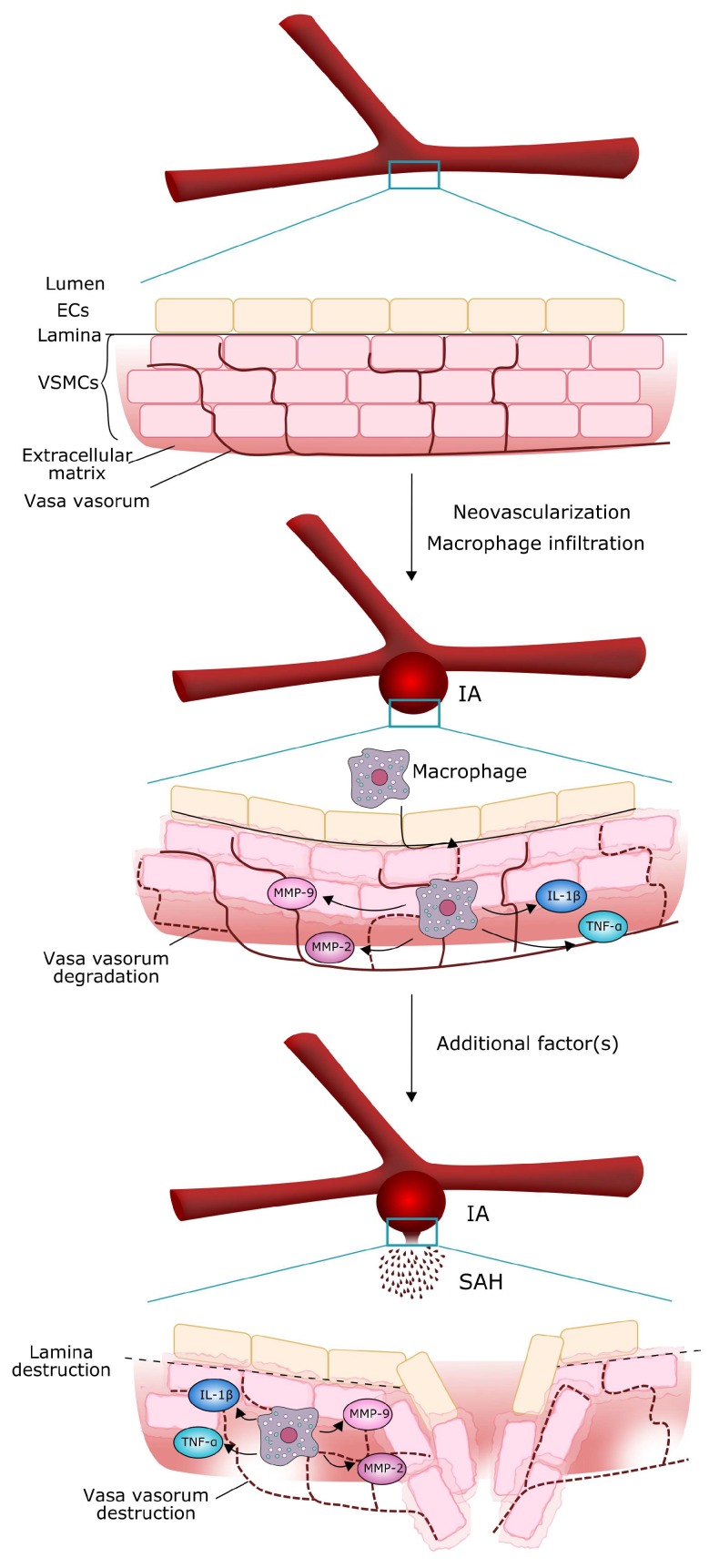
Molecular mechanism of intracranial aneurysm (IA) growth and rupture. Neovascularization (dashed thin red lines) of the vasa vasorum (solid thin red lines) in vascular smooth muscle cells (VSMCs) enables macrophages and other inflammatory cells to penetrate the vessel wall through the layer of endothelial cells (ECs) and secrete substances, mostly proteases, thus thinning and weakening the wall. Such structure of the vessel can rupture, resulting in subarachnoid hemorrhage (SAH), but a trigger, mostly unidentified, is needed to initiate this effect.

The development and rupture of IA depend on the remodeling of vessel walls, which, in turn, depends on the balance between matrix degeneration and regeneration. The former is executed by proteases, released by macrophages and other inflammatory cells, the latter is provided by smooth muscle cells. Inflammatory cells can gain access to the walls of affected vessels through a channel formed by the neovascularization of the vasa vasorum [12]. Therefore, angiogenesis, providing new vasa vasorum in cerebral artery wall, can be an early step during IA formation [3,12]. These new blood vessels can be infiltrated by inflammatory cells, mainly macrophages and neutrophils, which can remodel the wall to form a clinically detectable IA [13]. Further action of these cells secreting metalloproteinases and other remodeling enzymes, along with yet non-identified factors, can lead to IA rupture, resulting in SAH [13,14,15,16,17,18] (Figure 1). A comparison of the walls originating from ruptured and unruptured IA revealed a greater invasion of inflammatory cells in the former than in latter [19].

**Figure 2:**
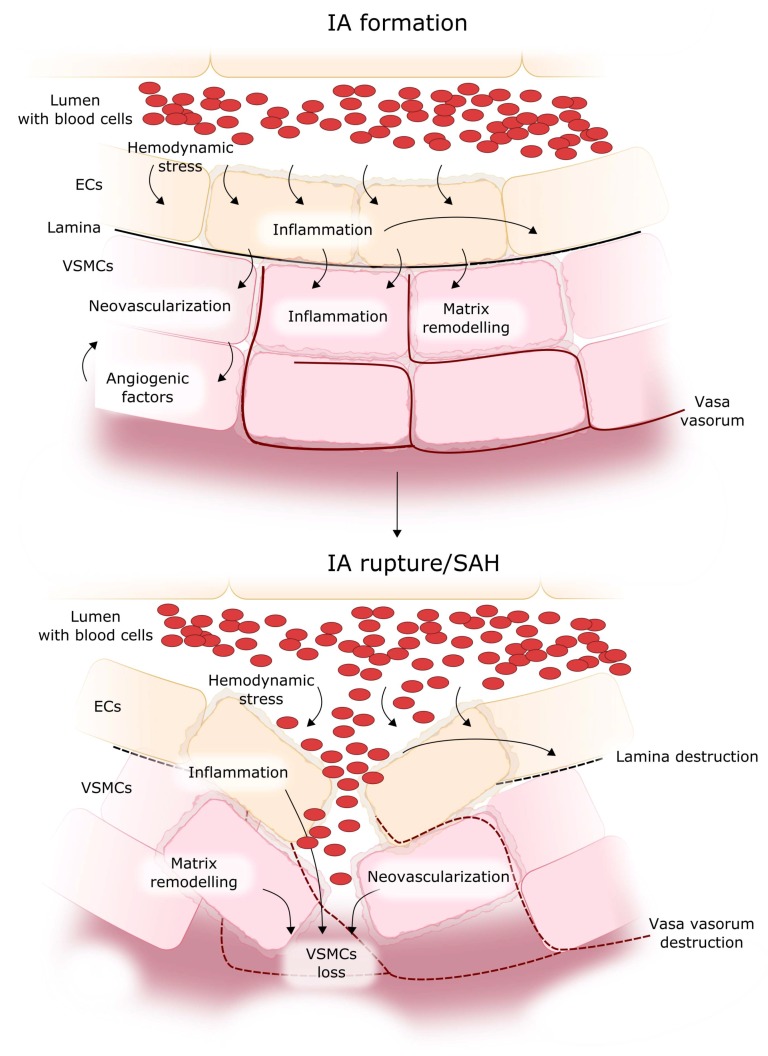
Mechanism of IA formation, progression, and rupture. Hemodynamic stress in a blood vessel stimulates inflammation in the endothelial cells (ECs), which promotes lamina degradation and evokes changes in vascular smooth muscle cells (VSMCs), including inflammation, neovascularization and matrix remodeling, which together modify the properties of the blood vessel wall. Permanent inflammation leads to the degeneration of VSMCs, resulting in thinning of the VSMC layers, increase in their fragility, and finally in IA rupture. Red dashed lines denote vasa vasorum, black dashed lines: destroyed lamina, red ovals represent blood cells.

Although our knowledge of IA pathogenesis is far from completeness, it is established that inflammation plays an important role in it [1,20,21]. The formation of IA is initiated by a hemodynamically triggered endothelial cell dysfunction followed by an inflammatory reaction with elevated activity of the inflammatory transcription factor NF-κB [22] (Figure 2). NF-κB denotes a family of transcription factors playing an important role in many cellular activities, including proliferation, response to stress, immune response, apoptosis, and inflammation. In humans, there are at least five members of the NF-κB family: NF-κB1 (p50), NF-κB2 (p52), RelA (RELA proto-oncogene, NF-κB subunit, p65), RelB (RELB proto-oncogene, NF-κB subunit), and c-Rel (REL proto-oncogene, NF-κB subunit) [23]. In normal condition, NF-κB dimerizes and is sequestered in the cytoplasm by its inhibitor IκB (nuclear factor of kappa light polypeptide gene enhancer in B cells inhibitor), which prevents the nuclear translocation of the dimer [24]. In stress conditions, NF-κB translocates to the nucleus, where it stimulates or represses the transcription of many genes.

**Figure 3:**
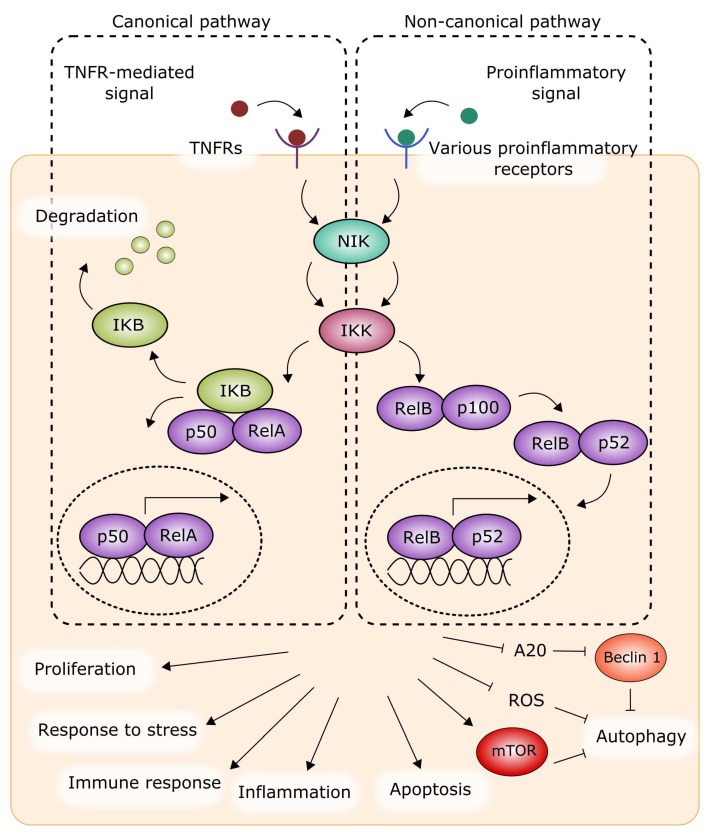
NF-κB (nuclear factor kappa-light-chain-enhancer of activated B cells) can be activated in a canonical (left) or non-canonical (right) pathway to stimulate or repress the expression of genes involved in the regulation of cellular response to stress, immune response, and inflammation. Either pathway is initiated by a ligand bound by its receptor. Some autophagy proteins can be related to both pathways, as discussed in Section 4. This figure is a simplified representation only and does not contain many important details. IKB (nuclear factor of kappa light polypeptide gene enhancer in B cells inhibitor); IKK: IκB kinase; mTOR: mammalian target of rapamycin; NIK: NF-κB-inducing kinase; TNFR: tumor necrosis factor receptor; ROS: reactive oxygen species. Subunits of NF-κB are shaded with the same, light fiolet color. Sharp arrows represent stimulation/induction, while blunt arrows: inhibition/downregulation, dashed oval: the nucleus.

The activation of NF-κB in basal (normal) conditions can be promoted by two separate signaling pathways, i.e., the canonical and non-canonical pathways, which can function in basal and stress conditions [25,26,27]. The basal canonical pathway is mediated by the inhibitor of the IκB kinase (IKK), which inactivates NF-κB [28,29]. Upon phosphorylation and degradation of IκB with the involvement of NF-κB-inducing kinase (NIK), the NF-κB heterodimer in the form of RelA/p50 is activated and translocates to the nucleus to control the transcription of assorted genes. The basal non-canonical pathway is mediated by NIK and IKK, which proteolytically process the protein p100 to produce p52, which forms a heterodimer with RelB, regulating gene transcription (Figure 3). In basal conditions, the signal sent by NIK is weak, as NIK is permanently being degraded by E3 ubiquitin ligases [30]. These basal pathways can be stimulated by various factors, but in general the canonical pathway is activated by tumor necrosis factor α (TNFα) and interleukin 1β (IL-1β), as well as other cytokines, with the involvement of their specific receptors [31]. The non-canonical pathway is activated with the contribution of a limited set of ligands, including those belonging to the TNF family and TNF-related factors [32,33].

Elevated activation of NF-κB leads to the expression of several pro-inflammatory proteins, including cyclooxygenase-2 (COX-2), prostaglandin E2 (PGE-2), and molecules which facilitate the recruitment and adhesion of macrophages, which enter the vessel wall through new vasa vasorum [15,34]. They secrete further pro-inflammatory molecules and proteins, mainly proteases, which can remodel the structure of the wall [35,36,37]. The inflammatory response in the vessel wall leads to a disruption of the internal elastic lamina and to the proteolytic destruction of the vascular extracellular matrix by matrix metalloproteinases (MMPs), with subsequent IA formation via the upregulation of other proteinases and angiogenic factors [38]. The inflammatory response also stimulates the phenotypic modulation of vascular smooth muscle cells (VSMCs) from a contractile to a pro-inflammatory/pro-matrix remodeling phenotype, followed by their degeneration, which may be crucial to IA formation and progression [39]. The phenotypic modulation of VSMC is associated with the expression of pro-inflammatory, pro-matrix remodeling genes. The expression of MMPs, which are products of such genes, is significant in both leukocytes and VSMC within the vessel/aneurysm wall [40]. MMPs digest the extracellular matrix in the arterial wall and cause further vessel damage. Subsequent changes in the media layer include apoptosis/loss of VSMC, changes in VSMC proliferation, and thinning of the aneurysm wall [41]. The resulting cell death and vessel wall degeneration ultimately culminate in IA rupture. Therefore, the rupture of IA results from a chain of molecular events producing changes in the brain vessel wall (Figure 2). These changes may be modulated by occasional endogenous or environmental influences but, basically, they are consequences of a chain of ordered events. In other words, the act of IA rupture can be triggered by an accidental affair, but only after the occurrence of some molecular events that are necessary for the susceptibility of IA to rupture.

As mentioned, the inflammatory cells find their way to a cerebral aneurysm through the vasculature of vasa vasorum. The primary source of these cells is not precisely known. It is possible that macrophages and other inflammatory cells stimulate neoangiogenesis of vasa vasorum. Proinflammatory factors released by inflammatory cells weaken the wall of the vessel degrading the extracellular matrix, which can lead to the breakage of the integrity of the vessel lumen [14]. The critical role of macropahges and other inflammatory cells in aneurysm formation and development was confirmed in many studies performed on animal models of IA as well as human ruptured and unruptured IAs [13]. Furthermore, prostaglandin E2–EP2–NF-κB signaling in macrophages is considered as a target in IA therapy [42].

## 3. Inflammation in Intracranial Aneurysm

The potential role of inflammation in the formation and progression of IA was proposed in several studies [43]. Macrophages are thought to be the key inflammatory component that infiltrate the walls of IA. Although macrophage infiltration was observed in both ruptured and unruptured IAs, the intensity of this process seems be associated with the loss of smooth muscle cells and the disruption of the collagen layer in the IA wall [19]. In addition, it was shown that the incidence of IAs formation was significantly decreased in macrophage-depleted mice [44]. These data suggest that the formation and rupture of IAs is related to macrophages accumulation.

T lymphocytes are other inflammatory cells detected in IA walls [19]. A positive correlation was found between the number of T cells and IA rupture. T lymphocytes actively participate in the inflammatory reaction, but it is still unclear whether their increased infiltrations is the cause or a consequence of the rupture.

**Figure 4:**
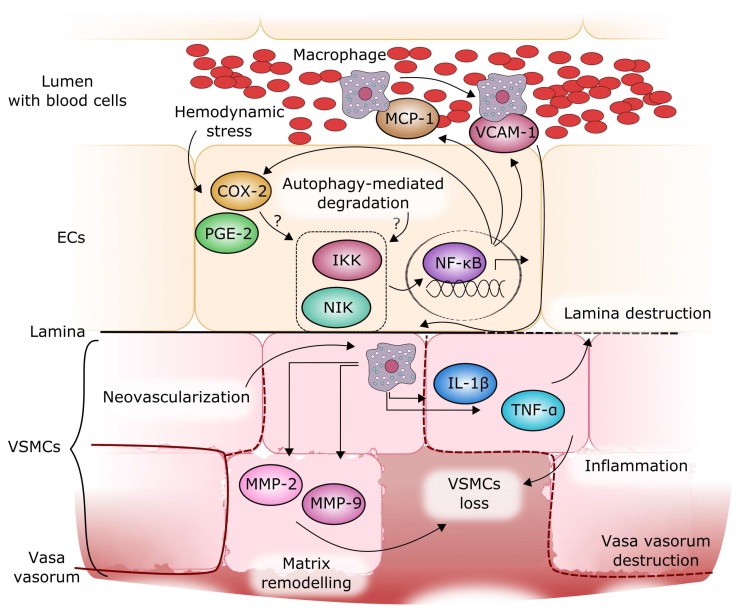
Molecular mechanism of intracranial aneurysm (IA) formation and progression. A wall shear stress can be an initial trigger in IA formation. This stress can result in some fragile sites (Figure 1) to which macrophages can be recruited by MCP-1 (monocyte chemoattractant protein 1), which is expressed after NF-κB activation, resulting also in the expression of other pro-inflammatory proteins, including COX-2 and PGE-2, in endothelial cells (ECs). Macrophages, which adhere to ECs with the involvement of VCAM-1 protein, find their way to the vessel wall through new vasa vasorum, secrete other pro-inflammatory proteins, including TNF-α, IL-1β, and matrix remodeling proteins such as matrix metalloproteinases MMP-2 and MMP-9. NF-κB activation is mediated by IKK (IκB kinase) and NIK (NF-κB-inducing kinase) proteins, whose activity is regulated by autophagy. The concerted action of these proteins can result in a further weakening of the wall structure and, eventually, in its rupture, but the action of some, yet unidentified factors, can be the final trigger of IA rupture. Black and red solid/dashed lines denote normal/disrupted lamina and vasa vasorum, respectively.

NF-κB has been identified as a regulatory transcription factor of genes induced in IA lesions and as a possible factor related to IA rupture [40] (Figure 4). Moreover, shear stress—an initiator of IA formation—was demonstrated to induce the expression of pro-inflammatory genes via an NF-κB-mediated pathway [45]. NF-κB over-activation leads to the expression of inflammatory proteins, including cyclooxygenase-2 (COX-2), prostaglandin E2 (PGE-2), and others, which stimulate IA progression [46]. NF-κB also induces the expression of MCP-1 (monocyte chemoattractant protein 1) and VCAM-1 (vascular cell adhesion molecule 1), responsible for the recruitment and adhesion of macrophages, which penetrate the vessel wall through junctions between endothelial cells and new vasa vasorum [15,34]. The macrophages release other pro-inflammatory molecules, including tumor necrosis factor α (TNF-α), interleukin 1β (IL-1β), metalloproteinases, as well as other proteases, which remodel the structure of the wall in concert with other molecules [35,36,37]. NF-κB was activated in endothelial cells and macrophages in IA walls during IA formation [47]. The deficiency or inhibition of NF-κB significantly reduced the expression and production of downstream pro-inflammatory factors and suppressed IA formation and progression in rodent models [46,48]. NF-κB is also a mediator of the downregulation of procollagen type I and III gene expression, which was observed in IA patients [46]. No mutation was found in type III procollagen gene in patients with IA, thus the reduced production of type III collagen may be derived from impaired NF-κB-dependent regulation of the expression of this gene. Several cytokines are associated with IA formation. Monocyte chemoattractant protein 1 (MCP-1) is a chemokine that modulates the migration and infiltration of monocytes and T lymphocytes. An increased expression of the *MCP-1* gene was found in the wall of IA at the early stage of its formation in rats [34]. Moreover, MCP-1 knockout mice showed a significant decrease in macrophage accumulation and a reduced incidence of IA, suggesting that MCP-1 may be a critical element in aneurysm pathogenesis [34]. VCAM-1 is other inflammatory component that participates in the recruitment of monocytes. It also regulates the adhesion of monocytes to endothelial cells in the vessel wall. An increased expression of VCAM-1 in human IA cases was described [49]. Cytokines, including MCP-1, SDF-1 (C-X-C motif chemokine ligand 12) and VCAM-1 are responsible for the recruitment of macrophages into the wall, thus they may initiate the inflammatory reaction and wall remodeling. Therefore, it seems that cytokines play a key role in aneurysm formation and progression, and their action can lead to the activation of NF-κB (Section 2 and Figure 2).

Although the role of inflammation in IA development is not fully understood, the above findings suggest that NF-κB can be crucial to aneurysm formation and progression. The inflammatory response involving leukocytes and cytokines seems to be associated with functional and morphological changes in the vessel wall that lead to IA.

Because of a low availability of human IA specimens, the studies on IA pathogenesis has been mainly conducted on animal models of IA, which strongly support a crucial role of the inflammatory response in the intracranial arterial walls in IA formation and progression. Among the factors regulating inflammatory processes, NF-κB can be one of the most significant proteins in the pathogenesis of IA formation and rupture [41].

## 4. NF-κB Signaling Pathways and Autophagy

Besides acting as a transcription factor, NF-κB can modulate several signaling pathways. The level of autophagy is controlled positively or negatively by NF-κB through its inducers. NF-κB activation by TNF inhibits autophagy by the activation of the mTOR (mechanistic target of rapamycin kinase) complex and the inhibition of reactive oxygen species (ROS) [50]. TRAF6 (TNF receptor-associated factor 6), a member of the TRAF family, can act as a signal transducer in the NF-κB signaling pathway [51]. It can also induce ubiquitination of Beclin 1, a key protein of autophagy, if stimulated by toll-like receptor 4 (TLR4) [52]. However, Beclin 1 ubiquitination is repressed by A20, an NF-κB inhibitor, resulting in autophagy inhibition [53]. Autophagy can be induced in hypoxic conditions by hypoxia-induced factor 1α (HIF-1α), but this induction is related to the activation of stress-responsive transcription factors, including NF-κB [54]. It was shown that autophagy activation by NF-κB was essential for the cell to survive after heat shock [55]. Moreover, the modulation of autophagy by NF-κB played a role in cell survival in response to protein aggregation stress, and this reaction seemed to be independent of the primary stress factor [56,57].

RelA(p65), a member of the NF-κB family, can target several sites in the promoter of the *BECN1* (*Beclin 1*) gene, modulating the level of autophagy in various human cell lines [58,59]. In turn, other members of the NF-κB family can be activated by DNA damage with the involvement of autophagy, as reported for many cell lines and several signaling proteins [60,61,62,63,64,65]. Bcl-2/E1B-19K interacting protein 3 (BNIP3) is a mitochondrial protein cooperating with several anti-apoptotic proteins and inducing cell death with the involvement of autophagy [66,67]. RelA(p65) was reported to block the binding of the BNIP3 promoter, and this effect also can be linked with a mutual control of NF-κB-directed inflammation and autophagy [68,69]. Transforming growth factor-β-activating kinase 1 (TAK1) can activate autophagy by the inhibition of mTORC1 (mechanistic target of rapamycin complex 1), in an analogous fashion to IKKs in starved cells [54,70]. However, it is not completely clear whether NF-κB is needed for such TAK1 action [71].

**Figure 5:**
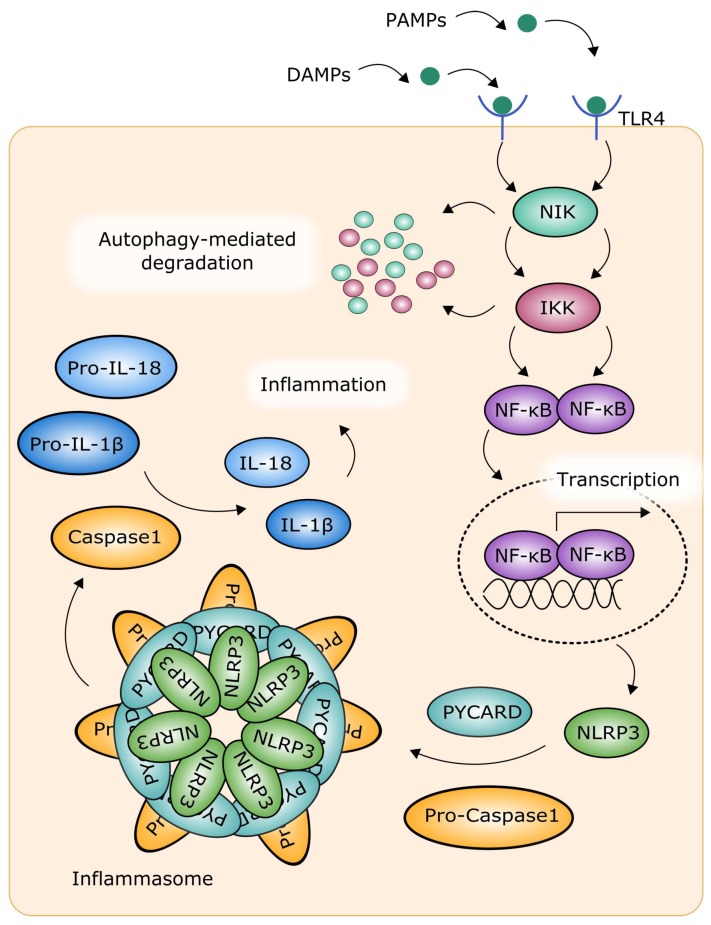
The inflammasome and the interplay between autophagy and NF-κB-mediated inflammation. See the text for more details. PYCARD (apoptosis-associated speck-like protein containing a caspase recruitment domain); DAMP: damage-associated molecular pattern; IKK: IκB kinase; IL: interleukin; NF-κB: nuclear factor kappa-light-chain-enhancer of activated B cells; NIK: NF-κB-inducing kinase; NLRP3: NLR family pyrin domain containing 3; PAMP: pathogen-associated pattern; TLR4: Toll-like receptor 4.

The inflammasome is a large signaling complex responsible for the activation of inflammatory processes. It can be formed in response to infection, disturbed metabolism, and tissue damage (Figure 5). The inflammasome contains caspase-1, which is an inflammatory protease involved in pyroptosis, a variant of programmed cellular death, and proteolytic cleavage of two inflammatory cytokines, namely, IL-1β and interleukin 18 (IL-18) [72]. The inflammasome can be formed in response to the cytosolic recognition of DAMPs (damage-associated molecular patterns) and PAMPs (pathogen-associated patterns) by nod-like receptors (NLRs) [73]. The inflammasome contains also PYCARD (apoptosis-associated speck-like protein containing a caspase recruitment domain), which is essential for its activation. Probably the best studied and known inflammasome is the NLRP3 (NLR (nucleotide-binding oligomerization domain, leucine rich repeat) family pyrin domain containing 3) inflammasome [74,75]. The activity of NLRP3 is induced by a variety of factors, including potassium ions, cholesterol, mitochondrial ROS, amyloid, fatty acids [76]. The NLRP3 inflammasome is primed by TLR signaling pathway with the involvement of NF-κB [77]. Autophagy can regulate the inflammasome by a mechanism dependent on NLR, controlling the level of inflammatory cytokines, IL-1β and IL-18 [78,79,80]. Autophagy can degrade NF-κB signaling proteins IKK and NIK, and this process is mediated by the inhibition of Hsp90 (heat shock protein 90) protein [81]. In general, autophagy can specifically degrade NF-κB signaling components, and this effect can result in the cessation of NF-κB activation or in its stimulation, when signaling inhibitors are degraded [71].

It was shown that inhibition of NF-κB could stimulate autophagy via the JNK (c-Jun N-terminal kinase) signaling pathway in porcine granulosa cells [82]. Activation of the JNK pathway and overexpression of Beclin 1 were reported in autophagy induced by ceramide in human hepatoma Hep3B cells [83].

## 5. Autophagy and Its Potential Involvement in Intracranial Aneurysm and Aneurysmal SAH

Autophagy (self-eating) in its basic form is a process of lysosomal degradation of cellular components which are no longer needed or are damaged. This degradation can yield elements which can be used by the cell to produce energy and/or cellular building blocks. Autophagy can be stimulated by different stimuli, including starvation. Therefore, autophagy can be considered as an important element in cellular metabolism, as well as a part of cytoplasmic quality control. These basic functions of autophagy can be extended by its action against external invaders, the regulation of programmed cellular death, its involvement in DNA damage response, and others [84]. Disturbed autophagy can be associated with human disorders, including cancer, immunological and neurodegenerative diseases, eye defects, and many others [85].

Autophagy can be divided into three classes. Macroautophagy helps the cell to survive in unfavorable energetic and/or nutritional conditions. The material to be degraded (cargo) is packaged into a membraned structure, the autophagosome, which is then transported into lysosomes for degradation. In microautophagy, the cargo is directly engulfed by the lysosomes. In chaperone-mediated autophagy, proteins with a specific amino acid motif, with the involvement of HSC70 (heat shock protein family A (Hsp70) member 8) protein, a chaperone, and several of its partners, are degraded.

**Figure 6:**
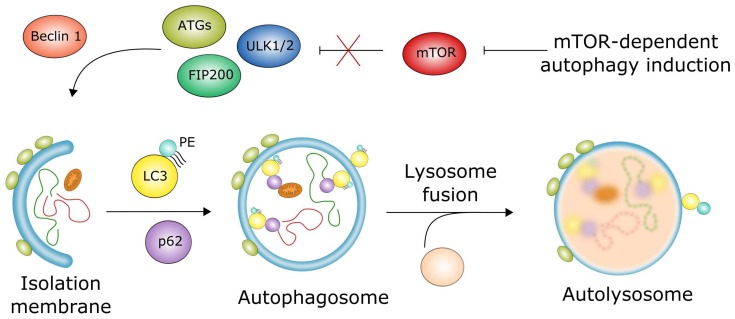
Macroautophagy dependent on mTOR (mammalian target of rapamycin) can be induced by a lack of nutrients, which leads to the activation of the ULK1 complex, containing UKL1/2, FIP200, ATG13, and ATG101, that is inhibited by the mTOR complex in normal conditions. This complex, with the involvement of Beclin 1, supports the formation of an isolation membrane (phagophore), which encircles the cargo, consisting of damaged and no longer needed cellular components, to form an autophagosome, a double-membrane vehicle which encloses the cargo. The process of autophagosome formation is mediated by several proteins, including LC3, and p62 (SQSTM1), which are essential for autophagy, and involves phospholipids, including phosphatidylethanoamine (PE). The autophagosome fuses with the lysosome forming the autolysosome, in which degradation and recycling of the cargo occurs. Sharp arrows denote stimulation/induction, while blunt arrows: inhibition/downregulation; an X mark denotes abolishing of the inhibitory action of mTOR.

Macroautophagy, further referred to as autophagy, requires a joint action of autophagosome and lysosome to degrade and recycle the cargo. Lysosomal degradation occurs with the involvement of many autophagy-related proteins (ATGs). Autophagy can be categorized on the basis of pivotal signaling pathways involved in its regulation—in general, as mTOR (mammalian target of rapamycin)-dependent and -independent [86]. In the former, the inactivation of mTOR is the decisive event opening the pathway to form an isolation membrane that will encircle the cargo, thus forming the autophagosome, which fuses with the lysosome to produce the autolysosome, in which the recycling of the cargo occurs (Figure 6). This chain of events is supported by a number of proteins undergoing activation and modifications. These include ATG13, ATG101 serine/threonine kinases (ULKs), LC3 (microtubule associated protein 1 light chain 3α), p62 (SQSTM1, sequestosome 1), and the main phospholipid used to build the autophagosome, phosphatidylethanoamine (PE). mTOR-independent autophagy in mammals was firstly described by Sarkar et al., who showed that lithium induced autophagy mediated by the inhibition of inositol monophosphatase and resulting in the lowering of intracellular inositol or inositol 1,4,5-trisphosphate (IP3) levels [87]. Many other small molecules were reported to induce autophagy in an mTOR-independent fashion [86,88].

**Figure 7:**
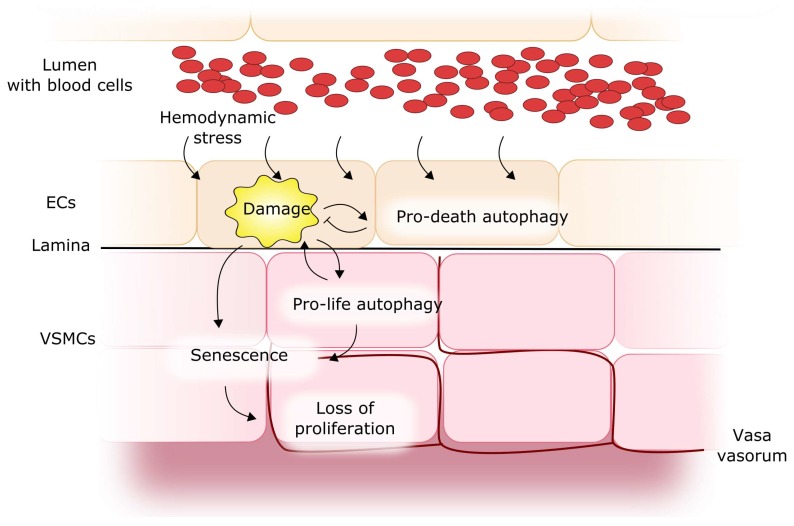
Autophagy in cerebral vessel walls. Damage to endothelial cells (ECs), following a shear stress, can result in pro-death autophagy activation, which increases the consequence of the damage because of a proapoptotic effect and other mechanisms. In addition, disturbances in pro-life autophagy may support the premature senescence of vascular smooth muscle cells (VSMCs), leading to their inability to replace damaged cells.

As mentioned, autophagy can be involved in the pathogenesis of many human diseases. However, its involvement in IA and SAH is a matter of debate. To consider the potential of autophagy in IA/SAH, it is reasonable to explore the role of autophagy in normal vessels, as basal autophagy is an important mediator of normal vascular function and it can play a protective role against damage to the vessel wall cells, including endothelial and smooth muscle cells [89,90]. However, such role of autophagy can be performed by proapoptotic mechanisms. Autophagy can be involved in the proliferation and acquisition of a synthetic phenotype by smooth muscle cells [91,92,93]. Research on experimental animals showed that defective autophagy in smooth muscle cells could support premature senescence in response to a stress factor [94]. Therefore, it is tempting to speculate that impaired autophagy can contribute to a senescent phenotype in the arterial walls, corresponding to aged vessels, in which the ratio of pathological events is higher (Figure 7). This could be seen in a wider context, exemplified by work showing that impaired autophagy can alter the morphology of vessel smooth muscle cells and impact their structural and functional properties. Rapamycin and its analogs, which are potent inducers of autophagy, are undergoing a number of clinical trials in vascular diseases, and stents coated with these drugs have revolutionized interventional cardiology [95].

As basal autophagy is a mechanism to survive for vessel smooth muscle cells, its stress-induced disturbance can result in the death of these cells, which, in turn, can lead to the weakening of the mechanistic properties of the wall and to its local rupture. This would explain the contribution of impaired autophagy to SAH resulting from IA rupture. However, impaired autophagy can be replaced by other protective mechanism(s), and this replacement could result in an increased protection, as shown by Grootaert et al. [94]. In that work, mice with a double deletion of the *ATG7* gene, essential for basal autophagy, were more resistant to oxidative stress than their wild-type counterparts. This effect was underlined by the stimulation of phase II enzymes GSTA (glutathione *S*-transferase) and NQO1 (NAD(P)H quinone dehydrogenase 1) by the SQSTM1 protein, overexpressed in cells with disturbed autophagy [96].

It was shown that SAH induced mitochondrial dysfunction, which, in turn, activated autophagy [97]. Therefore, targeting autophagy can lead to a decrease in SAH-related mitochondrial impairment, as shown with Epigallocatechin-3-gallate, a tea catechin. However, it cannot be definitely concluded that the mechanism of autophagy was a primary target of this catechin, or even that autophagy played a significant role in the observed effect.

**Figure 8:**
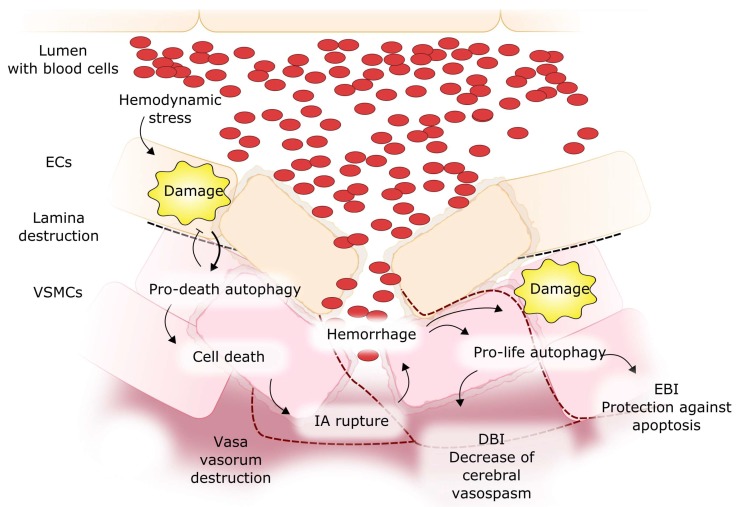
Involvement of autophagy in subarachnoid hemorrhage (SAH) resulted from rupture of an intracranial aneurysm and its consequent brain injury. Impaired pro-life autophagy can lead to the expansion of damage in endothelial cells (ECs) and vascular muscle smooth cells, by supporting the senescence of these cells and an antiapoptotic effect, which can lead to early brain injury (EBI). Pro-death autophagy can lead to cell death and thinning of EC and VSMC layers and thus participate in IA rupture. Cerebral vasospasm can be associated with delayed brain injury (DBI) after SAH, and autophagy can reduce this effect. Red dashed lines: vasa vasorum, black dashed lines: destroyed lamina.

The presence of blood in the subarachnoid space after SAH leads to several pathological effects accounting for brain injury, which can be generally divided into early brain injury (EBI) and delayed brain injury (DBI) [98]. A direct link between SAH and autophagy can result from the devastating effect of this kind of hemorrhage on single cells, as their organelles can be damaged in the affected regions of the brain [99] (Figure 8). In animal models of SAH, the activation of autophagy with rapamycin decreased the translocation of Bax, a proapoptotic protein, from the cytosol to the mitochondrial membranes, suggesting an anti-apoptotic effect of autophagy in EBI [100]. This was confirmed in research in which 3-methyladenine, an autophagy inhibitor, induced apoptosis and worsened the neurological deficit in the rat endovascular perforation SAH model [101]. There are more examples of the involvement of autophagy in SAH consequences, strongly suggesting the protective action of autophagy against EBI induced by SAH (reviewed in [8]). DBI after SAH can be associated with cerebral vasospasm, which is a highly devastating condition in IA patients who experienced SAH [102]. Autophagy was shown to be activated by cystatin C in a rat SAH model and decrease the degree of cerebral vasospasm after SAH induction, and the mechanism of this definitely profitable effect of autophagy in SAH-related complications is largely unknown [103]. It should be stressed that, although IA formation, SAH, EBI, cerebral vasospasm, and DBI may form a chain of ordered events, they do not always occur together, as relatively few IAs result in SAH and so in SAH-related brain injuries.

Transmembrane 9 superfamily 1 (TM9SF1) was identified as an autophagy-induced protein, which colocalized with LC3 and increased the number of autophagosomes in the cells [104]. This protein is essential for autophagy, as its knockdown resulted in an inhibition of autophagy induced by starvation. Wang et al. identified proteins which are differentially expressed in ruptured IA [105]. They observed a near 9-fold increase in the expression of TM9SF1 in samples of vessels from 14 patients with ruptured IAs and concluded that the formation and rupture of IA could be associated with autophagy, inflammation, and immune responses. However, those studies did not bring information on the mechanism of the involvement of TM9SF1 and autophagy in IA pathogenesis.

Reports on the association of IA with autophagy do not indicate an unequivocal relationship between beneficial and deleterious effects of autophagy activation and inhibition. Instead, a delicate balance between the extent of autophagy and IA and aneurysmal complications can be postulated, and further studies are needed to determine that extent.

Pompe disease is a rare, autosomal recessive disorder caused by defective acid α-glucosidase, with potential involvement of autophagy in its pathogenesis [106,107,108]. A late onset of this disease is frequently manifested by cerebrovascular complications, including aneurysm [109,110,111]. A case was reported where this kind of the disease presented with SAH following IA rupture [112]. Although the association between cerebrovascular complications and late-onset Pompe disease, as well as the involvement of autophagy in the pathogenesis of the disease are evident, they do not indicate directly a role that autophagy can play in IA. However, these results are a rationale for further research on the different phenotypes of Pompe disease to elucidate that role.

A high incidence of IA is also observed in autosomal dominant polycystic kidney disease (ADPKD), a genetic disease underlined by mutations in the polycystic disease 1 or 2 genes [113,114]. On the other hand, autophagy plays an important and emerging role in ADPKD pathogenesis [115,116]. Activators of autophagy were postulated to be potential therapeutic targets in ADPKD, as they suppressed cystogenesis in an ADPKD animal model [117]. However, no study associating ADPKD, IA, and autophagy has been performed so far, despite such a rationale.

## 6. Conclusions and Perspectives

The role of inflammation in IA has been appreciated in several reports, and emerging evidence suggests that this role can be critical in the pathogenesis of IA, its rupture, SAH, and their consequences—EBI, DBI, and cerebral vasospasm. NF-κB signaling can be the most important mechanism underlying this role. On the other hand, the possible involvement of autophagy in IA pathogenesis requires clarification, as some results suggest a direct involvement of this phenomenon in IA induction and development. However, there are many aspects of the interplay between NF-κB-mediated inflammation and autophagy, and both processes can be mutually dependent. Therefore, it can be speculated that some aspects of this interplay can be important in IA pathogenesis. This is also justified by the involvement of autophagy in normal vascular functions, including its protective role against damage to endothelial and smooth muscle cells. This role seems to follow from the general interplay between autophagy and apoptosis. Because disturbances in inflammatory or autophagic pathways or both are observed in many serious human diseases, information on the interaction between these pathways may be important for elucidating not only the molecular basis of human disorders, but also general cellular signaling pathways. From the clinical point of view, such information can be useful in the development of genetic profiles associated with an increased risk of disturbances of the mechanistic properties of cerebral arteries leading to their dysfunctions and resulting in IA; these profiles would be useful to identify individuals with an increased risk of IA rupture leading to SAH.

Autophagy can play an important role in IA-related SAH. However, this process seems to have a potential to help brain cells to survive after SAH; on the other hand, an extensive “self-eating” mechanism can result in cellular death. Therefore, the precise role of the balance between the pro-life and pro-death functions of autophagy in SAH consequences should be established. The relationship between these two faces of autophagy is a general problem in physiology and pathology, which awaits a definite solution and should be addressed in further research.

To explore further the role of the interplay between autophagy and NF-κB-controlled inflammation in IA/SAH, a good experimental model of these disorders is needed. There are many animal models of IA and SAH, and they can be further exploited by the use of animals with additional genetic modifications. These can include models expressing genes whose products are important for autophagy and NF-κB-directed inflammation. However, the existing animal models have been usually created with a specific factor of IA pathogenesis. Therefore, inclusion of an autophagy/NF-κB-element in those models will allow to assess it as a possible pathogenic/protective factor in IA. The formation and development of IA include a complex set of events, and we tried to show that that the NF-κB/autophagy interplay is worth further studies. Another experimental model can be considered, consisting of cerebral blood vessel cells obtained by differentiation of human induced pluripotent stem cells derived from individuals with genetic predisposition to IA, with a known history of the disease.

It should be taken into account that SAH can be a confounding factor in studies linking inflammation with IA pathogenesis. SAH can drive inflammatory mediators that may not be related to the processes occurring in the vessel walls but contribute to IA formation and progression as well as to SAH itself.

Although the involvement of autophagy in IA and its consequences was not evidenced in any patient study or histological examination, we have tried to present arguments showing that in vitro and animal model-based studies provide results that open a perspective for their translation into the clinic.

Finally, the question asked in the title of this review cannot be definitely answered now, but further studies to do so are justified. These studies can bring results important from a molecular biology point of view and which can also be exploited in IA prevention and therapy, as well as in the identification of IA patients with an increased SAH risk.
